# B-cell lymphoma 6 protein stimulates oncogenicity of human breast cancer cells

**DOI:** 10.1186/1471-2407-14-418

**Published:** 2014-06-10

**Authors:** Qiang Wu, Xue Liu, Hong Yan, Yin-huan He, Shan Ye, Xing-wang Cheng, Gui-lu Zhu, Wen-yong Wu, Xiao-nan Wang, Xiang-jun Kong, Xiao-chun Xu, Peter E Lobie, Tao Zhu, Zheng-sheng Wu

**Affiliations:** 1 Department of Pathology, Anhui Medical University, Hefei, Anhui, China; 2 Department of Emergency Surgery, The First Affiliated Hospital, Bengbu Medical University, Anhui, Bengbu, China; 3 Department of General Surgery, The First Affiliated Hospital, Anhui Medical University, Hefei, Anhui, China; 4 School of Life Science, Chinese University of Science and Technology, Hefei, Anhui, China; 5 Department of Clinical Cancer Prevention, The University of Texas MD Anderson Cancer Center, Houston, Texas, USA; 6 Cancer Science Institute of Singapore and Department of Pharmacology, National University of Singapore, Singapore, Singapore

**Keywords:** Breast cancer, BCL6, microRNA

## Abstract

**Background:**

B-cell lymphoma 6 (BCL6) protein, an evolutionarily conserved zinc finger transcription factor, showed to be highly expressed in various human cancers in addition to malignancies in the lymphoid system. This study investigated the role of BCL6 expression in breast cancer and its clinical significance in breast cancer patients.

**Methods:**

Expression of BCL6 protein was assessed using *in situ* hybridization and immunohistochemistry in 127 breast cancer patients and 50 patients with breast benign disease as well as in breast cell lines. Expression of BCL6 was restored or knocked down in two breast cancer cell lines (MCF-7 and T47D) using BCL6 cDNA and siRNA, respectively. The phenotypic change of these breast cancer cell lines was assessed using cell viability MTT, Transwell invasion, colony formation, and flow cytometry assays and in a xenograft mice model. Luciferase reporter gene, immunoblot, and qRT-PCR were used to investigate the molecular events after manipulated BCL6 expression in breast cancer cells.

**Results:**

BCL6 protein was highly expressed in breast cancer cell lines and tissue specimens and expression of BCL6 protein was associated with disease progression and poor survival of breast cancer patients. *In vitro*, the forced expression of BCL6 results in increased proliferation, anchorage-independent growth, migration, invasion and survival of breast cancer cell lines, whereas knockdown of BCL6 expression reduced these oncogenic properties of breast cancer cells. Moreover, forced expression of BCL6 increased tumor growth and invasiveness in a nude mouse xenograft model. At the gene level, BCL6 was a target gene of miR-339-5p. Expression of BCL6 induced expression of CXCR4 and cyclinD1 proteins.

**Conclusions:**

The current study demonstrated the oncogenic property of BCL6 in breast cancer and further study could target BCL6 as a novel potential therapeutic strategy for breast cancer.

## Background

Breast cancer is the most common worldwide malignancy in women, accounting for approximately 29% of new cancer cases annually in women in the United States [1]. Despite considerable advances in diagnostic and therapeutic approaches over the past decades, breast cancer is still the second most common cause of cancer death in women [1]. Better understanding of the molecular mechanisms and gene alterations in breast cancer could lead to more effective control of breast cancer clinically. To date, numerous tumor suppressor genes and oncogenes have been identified in breast cancer and further studies of these gene alterations and functions will assist in revealing the molecular mechanisms of breast cancer initiation and progression [2].

To this end, human B-cell lymphoma 6 (BCL6) is a 95 kDa nuclear protein, belonging to the BTB/POZ (BR-C, ttk and bab/Pox virus and Zinc finger) domain family of transcription factors. BCL6 protein has been reported as a master regulator of B lymphocyte development and growth [3,4] and altered BCL6 protein expression was implicated in pathogenesis of diverse human hematologic malignancies, especially in the diffuse large B cell lymphoma (DLBCL), the most common lymphoma in adults [5-7]. Overexpression of BCL6 was frequently shown in DLBCL patients due to a functional mutation in the BCL6 promoter [5]. BCL6 protein is a potent inhibitor of senescence of primary mouse embryonic fibroblasts and BCL6 expression also dramatically extends the replicative lifespan of primary human B cells [8]. Recently, BCL6 protein was also shown to be highly expressed in various human cancers other than malignancy in the lymphoid system. For example, Kanazawa et al. showed that BCL6 protein was expressed in normal epidermis and epidermal neoplasms, suggesting that expression of BCL6 protein may be associated with differentiation in normal and neoplastic epidermal cells [9]. Chamdin et al. reported that BCL6 was expressed in neuroblastoma, expression of which was significantly associated to poor survival of the patients [10]. In the mammary glands, BCL6 protein was expressed in the mammary epithelium in nonpregnant and early pregnancy animals [11] and overexpression of BCL6 prevented the duct formation and apoptosis in murine mammary epithelium [11,12]. However, BCL6 protein was overexpressed in breast cancer tissues, especially in high-grade ductal breast cancer compared to normal mammary gland tissues [12,13]. BCL6 expression was able to induce expression of tumor metastasis-related genes in breast cancer cell lines [14]. These data suggested that BCL6 may possess an oncogenic function in breast cancer development. However, contradicted data did show that BCL6 expression was inversely associated with breast cancer cell lymph node metastasis, but associated with survival of breast cancer patients [14]. Overall, the role of BCL6 protein in human cancers other than in the lymphoid system remains to be determined. Thus, in this study, we first determined expression of BCL6 protein in breast cancer tissues and cell lines, and then associated BCL6 expression with disease progression and prognosis. After that, we investigated the role of BCL6 expression in regulation of breast cancer cell proliferation, migration, invasion, and survival *in vitro* and in xenografts models. We also explored the underlying molecular events of BCL6 action in breast cancer cells.

## Methods

### Cell lines and culture

Human breast cancer MCF-7, T47D, SKBR3, MDA-MB-453, MDA-MB-435S, and BT549 cell lines, a human breast non-tumorigenic MCF-10A cell line, and a human mammary epithelial (HMEC) cell line were obtained from the American Type Culture Collection (ATCC, Manassas, VA, USA) and cultured under the ATCC-recommended conditions. All cells were maintained in a humidified incubator at 37°C and 5% CO_2_.

### Breast tissue specimens

In this study, we collected two different cohorts of human breast tissue specimens, i.e., for *in situ* hybridization and immunohistochemistry, we recruited 127 patients with breast cancer and 50 patients with breast benign disease who underwent surgical treatment at The First Affiliated Hospital, Anhui Medical University (Hefei, China) between 2003 and 2006; for qRT-PCR, fresh tissue specimens from 30 breast cancer and 25 breast benign disease patients were prospectively collected between 2010 and 2011 from the same hospital. The fresh tissue specimens were immediately placed in a cryovial after surgery, snap-frozen, and stored in liquid nitrogen until use. Breast cancer patients who had undergone chemotherapy or radiation therapy before surgery were excluded. All breast cancer patients were female and received radical mastectomy or modified radical mastectomy. These 127 breast cancer patients were followed-up for a median 60 months. A protocol to use patient samples was approved by the Biomedical Ethics Committee of Anhui Medical University and a written informed consent was obtained from each patient.

### *In situ* hybridization and immunohistochemistry

Formalin-fixed and paraffin-embedded tissue specimens from 127 breast cancer patients and 50 breast benign disease patients were used to construct tissue microarrays and cut into 4-μm-thick sections. For *in situ* hybridization, digoxin-labeled antisense oligonucleotide probes for BCL6 cDNA were obtained from Boshide Biotech Co. (Wuhan, China). The probe sequences were 5′-GACAGCTGTATCCAGTTCACCCGCCATGCCAGTGA-3′, 5′-TTCTATAGCATCTTTACAGACCAGTTGAAATGCAA-3′, and 5′-ATCCTGCAGATGGAGCATGTTGTGGACAGTTGCCG -3′.

For immunohistochemical analysis of BCL6 expression in tissue samples, a rabbit anti-BCL6 polyclonal antibody was obtained for Santa Cruz Biotechnologies (Santa Cruz, CA, USA) and used at a dilution of 1:100 according to our previous studies [15,16].

Expression of BCL6 mRNA and protein in breast tissue specimens were reviewed and scored by two pathologists (QW and ZSW) using a light microscope (Olympus) using the staining intensity and percentage of tissue staining, i.e., 10% percent or more tumor cells stained were considered as positive, whereas <10% tumor cells stained with any intensity was considered as negative.

### Plasmid constructions and generation of stable BCL6-expressing cell lines

The coding sequence of human BCL6 transcript variant 1 (GenBank accession #NM_−_001706) was cloned into a mammalian expression vector pReceiver (GeneCopoeia, Guangzhou, China) according to the manufacture’s protocol. After DNA sequence confirmation, this vector was named as pReceiver-BCL6 (BCL6). MCF-7 cells were then stably transfected with pReceiver-BCL6 or the empty pReceiver plasmids (VEC) to establish stable cells, MCF-7-BCL6, with forced expression of BCL6 and their control cells, MCF-7-VEC, respectively.

### Transfection of siRNA and miRNA

To knockdown BCL6 expression or manipulate miRNA expression, we choose T47D and MCF-7 cells as a pair of model cell lines for gene transfection. Briefly, cells (1.0 ×10^5^ /well) were seeded in 6-well plates and transiently transfected with BCL6 small interfering RNA (siRNA) or control scrambled siRNA duplex (GenePharma, Shanghai, China) or with 2’-O methylated single-stranded miR-339-5p antisense oligonucleotides (ASO) vs. its negative control or miR-339-5p mimics (all from GenePharma) vs. its negative control using Lipofectamine 2000 (Invitrogen, Carlsbad, CA, USA) according to the manufacturer’s instructions. The sequences of BCL6 siRNA and scrambled control siRNA duplex were listed in Additional file 1: Table S1.

### RNA isolation and quantitative polymerase chain reaction

Total cellular RNA was isolated using a Trizol reagent (Invitrogen) according to manufacturer’s instructions. qRT-PCR was then performed to detect expression of BCL6, GAPDH, miR-339-5p, U6, and common tumor-related genes as described previously [15,17,18]. The sequence of the primers used for qRT-PCR was summarized in Additional file 1: Table S2.

### Protein extraction and Western blot

Total cellular protein and western blot analysis were performed according to previous studies [15,19]. The antibodies used were as follows: a rabbit anti-BCL6 polyclonal antibody (Santa Cruz Biotechnologies), a mouse anti-cyclinD1 monoclonal antibody (Santa Cruz Biotechnologies), a rabbit anti-CXCR4 (Bioss, Beijing, China), and a mouse anti-GAPDH monoclonal antibody (Santa Cruz Biotechnologies).

### Assays for cell phenotypic changes

Cell phenotypic changes after gene manipulations included proliferation, soft agar colony formation, cell migration and invasion in MCF-7 and T47D/MDA-MB-453 cells and the corresponding assays were performed as described previously [15,17-19]. In addition, we performed the cell wound healing assay to analyze tumor cell migration capacity. Briefly, T47D cells were seeded into 6-well plates and transfected with a BCL6 siRNA or NC vector. Upon cells reached totally confluence, scratching was done using a plastic tip. The wounded monolayers were incubated at 37°C in 1640 containing 10% FBS with or without mitomycin C (10 μg/ml, Sigma, St Louis, MO, USA) to block mitosis. Photos were taken at different periods of time under a microscope and the wound healing after scratched was measured daily.

### Flow cytometry assay

Cell apoptosis was assayed using the Annexin V-Apoptosis Detection kit (BestBio, Shanghai, China) according to the manufacturer’s instructions. All the experiments were performed using a FACScalibur cytometer (BD Biosciences, San Jose, CA). Cell cycle distribution was analyzed using the PI method. Each experiment was performed in triplicate and repeated at least once.

### Nude mouse breast cancer cell xenograft assay

All animal work was performed according to the animal care and use regulations of Anhui Medical University with the approved protocol by Biomedical Ethics Committee of Anhui Medical University. Briefly, 5 × 10^6^ MCF-7-VEC and MCF-7-BCL6 cells were suspended in 120 μl Matrigel/PBS at a radio of 1:1 (v/v) and then injected into the mammary fat pad of female BALB/c-nu (Slaccas, Hunan, China). The day before injection, one estrogen pellet (17β-estradiol, 0.72 mg/pellet, Innovative Research of America, Sarasota, FL) was implanted into each mouse. Tumor growth was detected by measuring the tumor mass twice a week using a formula = (length x width^2^)/2. The mice were ultimately sacrificed on Day 27 after implantation. Primary tumors and tumors metastasized to other organs, such as the lung and liver, were collected for further analysis.

### Luciferase reporter assay

The 3’UTR region of BCL6 was cloned to the psiCHECK-2 vector, including luciferase reporter gene. BCL6 3’UTR was amplified with primers of 5’-CCAGCCACAAGACCGTCCAT-3′ and 5′-CTCCGCAGGTTTCGCATTT-3′ and then inserted into the XhoI and NotI sites of the psiCHECK-2 vector. A psiCHECK-2 construct containing 3’UTR of BCL6 with a mutated sequence of miR-339-5p was also generated. All constructs were verified by DNA sequencing. After that, psiCHECK-2-BCL6 3’UTR and psiCHECK-2-mut-BCL6 3’UTR were co-transfected with 20 pmol miRNA-339-5p mimics or its negative control into breast cancer cells using Lipofectamine 2000 as described previously [15,17]. Firefly luciferase activity was normalized to Renilla luciferase activity. All experiments were performed in triplicate and repeated twice.

### Statistical analyses

All statistical analyses were performed using SPSS software for Windows (version 13.0; SPSS, Chicago, IL, USA). Differences between groups were compared using Pearson’s chi-square test for qualitative variables and Student’s t-test for continuous variables. Kaplan-Meier curves were constructed to determine patient relapse-free survival (RFS) and overall survival (OS). The statistical differences in survival among subgroups were compared using the log-rank test. *P* value <0.05 was considered statistically significant.

## Results

### Increase in BCL6 expression in breast cancer cell lines and tissues

**Figure 1:**
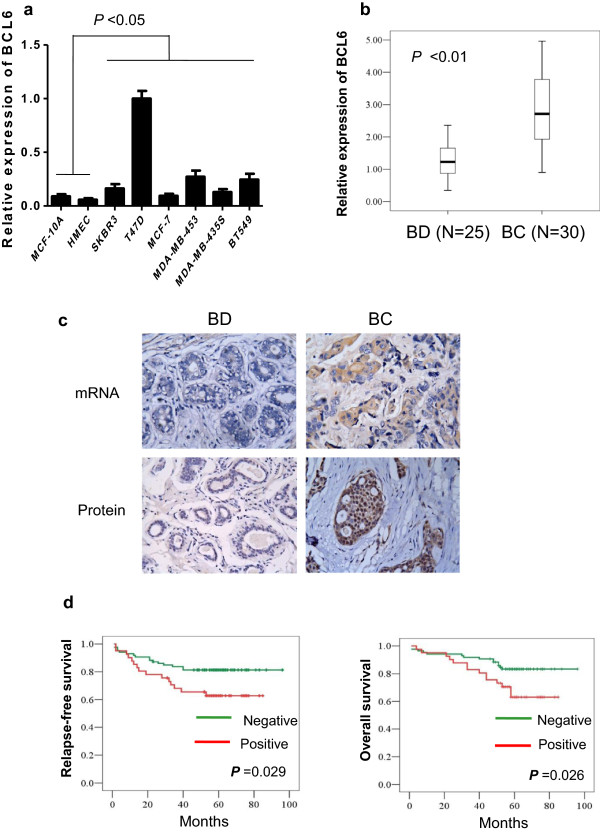
**Expression of BCL6 mRNA and protein in human breast cancer cell lines and tissue specimens. (a)** qRT-PCR. Level of BCL6 mRNA expression in eight human mammary cell lines was analyzed by qRT-PCR. **(b)** qRT-PCR. Levels of BCL6 mRNA expression were examined in 30 breast cancer (BC) and 25 breast benign disease tissue specimens (BD) by qRT-PCR. **(c)** Representative imagines of BCL6 expression analyzed by *in situ* hybridization and immunohistochemistry (Magnification: ×400). **(d)** Kaplan-Meier curve of the relapse-free survival (RFS) or overall survival (OS) according to BCL6 expression.

Expression of BCL6 mRNA in breast cancer and non-tumorigenic cell lines was analyzed by qRT-PCR and the data showed levels of BCL6 mRNA were significantly higher in breast cancer cell lines than in non-tumorigenic mammary epidermal cells (*P* < 0.05; Figure 1a). Similarly, BCL6 mRNA level was also significantly higher than in breast benign disease tissue specimens (*P* < 0.01; Figure 1b). After that, we confirmed these data in additional cohort of samples that included archival formalin-fixed paraffin-embedded breast tissue specimens from 127 breast cancers and 50 breast benign diseases using *in situ* hybridization and immunohistochemistry. As shown in Figure 1c and Table 1, expression of BCL6 mRNA and protein was significant higher in breast cancer tissues than in breast benign disease tissues (both *P* < 0.01).

**Table 1:** Expression of BCL6 in breast cancer and benign breast disease tissues

		**BCL6 mRNA**	**BCL6 protein**
**Group**	***n***	**Positive, *n * (%)**	**Positive, *n* (%)**
Benign breast disease	50	5 (10.0)*	3 (6.0)*
Breast cancer	127	68 (53.5)	41 (32.3)

Note: * P <0.01.

### Association of BCL6 protein expression with clinicopathological and survival data from breast cancer patients

We then associated BCL6 expression with clinicopathological features and survival of breast cancer patients and found that expression of BCL6 protein was positively associated with tumor size (*P* = 0.004), higher tumor grade (*P* = 0.003), tumor lymph node metastasis (*P* = 0.029), advanced clinical stages (*P* = 0.006) and Ki67 labeling index (*P* = 0.002) of breast cancer (Table 2). Kaplan-Meier analyses showed that patients with BCL6 protein-negative primary tumors exhibited higher five-year overall and disease-free survivals than patients with BCL6 protein-positive tumors (*P* = 0.026 and 0.029, respectively; Figure 1d).

**Table 2:** Association of BCL6 protein expression with clinicopathological parameters from breast cancer patients

**Parameter**	***n***	**BCL6 protein**	
		**Positive**, ***n *** **(%)**	***P *** **value**
Age (years)			
≤ 35	6	2 (33.3)	0.253
35-55	84	31 (36.9)	
> 55	37	8 (21.6)	
Tumor size (cm)			
≤ 2	44	7 (15.9)	**0.004**
>2	83	34 (41.0)	
Lymph node metastasis			
No	58	13 (22.4)	**0.029**
Yes	69	28 (40.6)	
Grade			
I	28	4 (14.3)	**0.003**
II	75	23 (30.7)	
III	24	14 (58.3)	
Stage			
I	28	3 (10.7)	**0.006**
II - III	99	38 (38.4)	
Estrogen receptor			
Negative	56	22 (39.3)	0.134
Positive	71	19 (26.8)	
Progesterone receptor			
Negative	64	22 (34.4)	0.611
Positive	63	19 (30.2)	
c-erbB-2			
low	93	32 (34.4)	0.397
high	34	9 (26.5)	
Ki67			
≤50%	50	8 (16.0)	**0.002**
>50%	77	33 (42.9)	

Values in bold are significant (P < 0.05).

### Expression of BCL6 promoted breast cancer cell proliferation, migration, and invasion, and inhibited apoptosis *in vitro*

**Figure 2:**
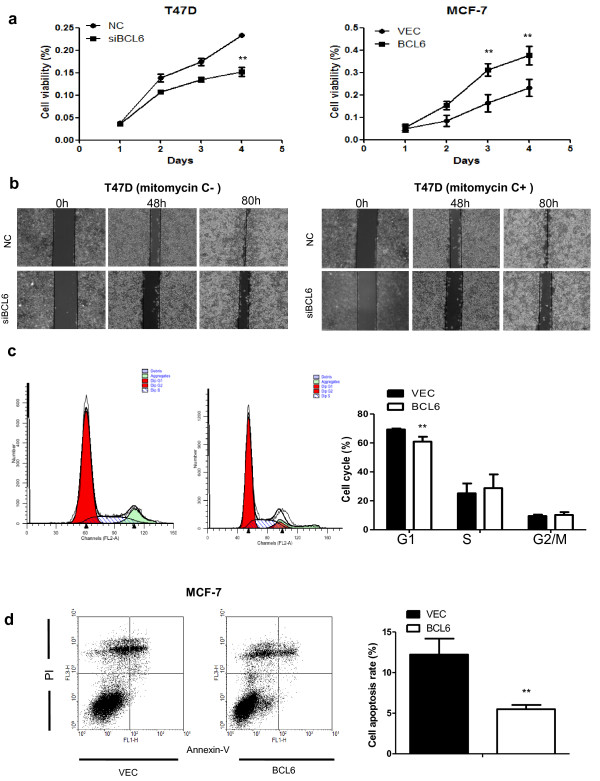
**Effects of BCL6 on regulation of breast cancer cell phenotype. (a)** Cell viability MTT assay. Cells were transiently transfected with BCL6 siRNA vs. negative control (NC) or BCL6 cDNA vs. control vector (VEC), respectively and then seeded in 96-well plates (3 × 10^3^ per well) and grown for 4 days for MTT assay. **(b)** Wound healing assay. T47D cells were grown and transiently transfected with BCL6 siRNA or negative control (NC), the wounded monolayers were cultured in the absence (left) or presence (right) of mitomycin C. **(c)** Flow cytometric analysis of cell cycle distribution in MCF-7 cells after gene transfection. **(d)** Flow cytometric analysis of apoptosis in MCF-7 cells after gene transfection. The average of apoptosis rate is presented as mean ± SD. All experiments were repeated at least three times. **P < 0.01.

We assessed the effects of forced expression of BCL6 on breast cancer cell growth, migration, and invasion *in vitro*. Based on the expression levels of BCL6 in breast cancer cell lines (Figure 1a), we therefore selected T47D and MCF-7 cells as model cell lines for the loss-of-function and gain-of-function analyses. BCL6 siRNA and cDNA was transiently transfected into T47D and MCF-7 cells, respectively. We observed that BCL6 siRNA decreased expression of BCL6 in T47D cells (Additional file 2: Figure S1a), whereas BCL6 cDNA transfection increased BCL6 expression in MCF-7 cells (Additional file 2: Figure S1b). Expression of BCL6 protein promoted MCF-7 cell viability (*P* < 0.01; Figure 2a, right), whereas depletion of BCL6 expression reduced T47D cell viability (*P* < 0.01; Figure 2a, left). Wound healing assays showed that BCL6 depletion leaded to slower closing of the scratch wounds in T47D cells compared with the control vector-transfected cells (Figure 2b, left). We also treated these cells with mitomycin C to block cell mitosis, which therefore allowed us to analyze cell migration in absence of cell proliferation. Our data revealed that treatment with mitomycin C did not affect the time course of wound closure, indicating that the effect of BCL6 depletion on cell migration was not dependent on cell proliferation (Figure 2b, right).

Furthermore, forced expression of BCL6 significantly increased the G2/M phase population in MCF-7 cells, but had more profound effect on G1 phase population with an 8.41% increase compared to the control (*P* < 0.01; Figure 2c). The increased BCL6 expression significantly reduced apoptosis of MCF-7 cells, with a 6.72% decrease compared with the control (*P* < 0.01; Figure 2d).

**Figure 3:**
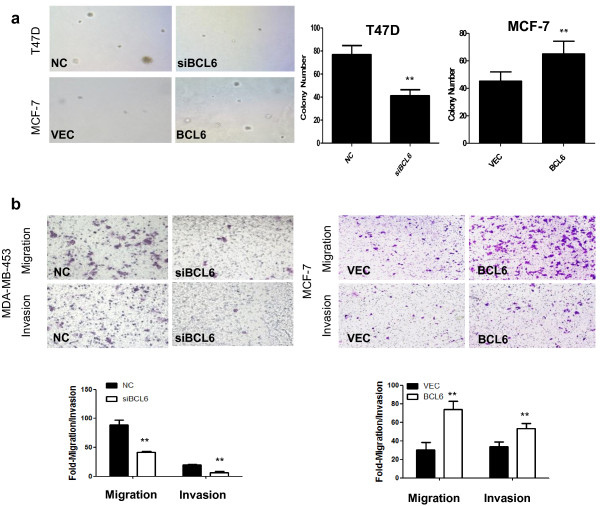
**Effects of BCL6 expression on regulation of breast cancer colony formation and migration and invasion capacity. (a)** Soft agar assay. After gene transfection, cells were seeded in 0.35% top agarose and 10% FBS in six-well plates in triplicate. The number of colonies was counted after 14 days incubation. **(b)** Tumor cell migration and invasion assay. MDA-MB-453 cells were grown and transiently transfected with BCL6 siRNA or negative control (NC) for 72 h. MCF-7 cells were grown and transiently transfected with BCL6 cDNA or vector-only (VEC) for 48 h. Cells in the upper chamber were removed and those cells migrated to the lower layer of the inner chamber were stained and counted. **, *P* < 0.01.

In addition, depletion of BCL6 expression significantly decreased colony formation of T47D cells by 46.4% compared to the control (*P* < 0.01; Figure 3a). In contrast, forced BCL6 expression significantly increased colony formation in MCF-7 by 30.0% compared to controls (*P* < 0.01; Figure 3b).

We next determined the potential impact of BCL6 on breast cancer cell migration and invasion capacity. Due to the weak invasive capacity of T74D cells, we choose another cell line with relatively high expression of BCL6, MDA-MB-453, to perform loss-of-function experiments in the Transwell assay. The number of migrated MDA-MB-453 cells was reduced to 53.2% after transfection with BCL6 siRNA (*P* < 0.01; Figure 3c), and was 66.9% lower than control cell for cell invasion (*P* < 0.01; Figure 3c). In contrast, forced expression of BCL6 protein increased the capacity of MCF-7 cell migration and invasion compared to the control cells (*P* < 0.01; Figure 3c).

### Expression of BCL6 promoted growth and invasiveness of MCF-7 cells in nude mouse xenografts

**Figure 4:**
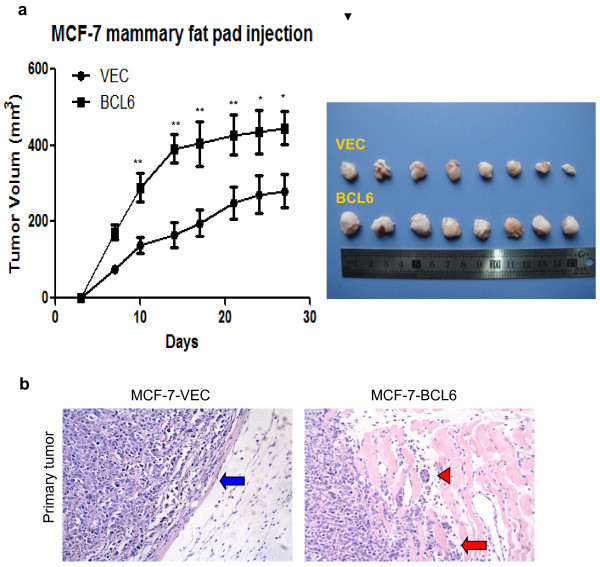
**Effects of BCL6 expression on regulation of MCF-7 xenograft growth in nude mice.** MCF7-VEC and MCF7-BCL6 cells were transplanted into the mammary fat pad of female BALB/c-nu, respectively. The volume of xenografts was measured twice a week and calculated. **(a)** Xenograft growth curve of MCF7-VEC and MCF7-BCL6-derived tumors over 27 days. **(b)** Hematoxylin and eosin staining of tumor xenograft sections. More aggressive behavior was observed in the margin of tumor nodule of MCF-7-BCL6 cells (red arrow) compared to that of MCF-7-VEC cells (blue arrow). Tumor embolus (red arrow head) was visualized in blood vessel (Magnification: ×200). *, *P* < 0.05; **, *P* < 0.01.

To further determine the effect of BCL6 expression in regulation of breast cancer proliferation and progression *in vivo*, we injected MCF-7-VEC and MCF-7-BCL6 cells orthotopically into the mammary fat pad of female BALB/c nude mice, respectively. The data showed both groups of cells formed palpable and measurable tumors, while MCF-7-BCL6 cell xenografts were significantly larger than those of MCF-7-VEC xenografts (*P* < 0.05; Figure 4a). Histology of xenografts showed that tumors derived from MCF-7-BCL6 cells were poorly encapsulated and highly invasive (Figure 4b). Interestingly, tumor cell emboli were observed in MCF-7-BCL6 xenografts but not in the control xenografts, suggesting that BCL6 expression potentially promoted tumor cell invasion and metastasis (Figure 4b).

### Expression of BCL6 increased expression of CXCR4 and cyclinD1

**Figure 5:**
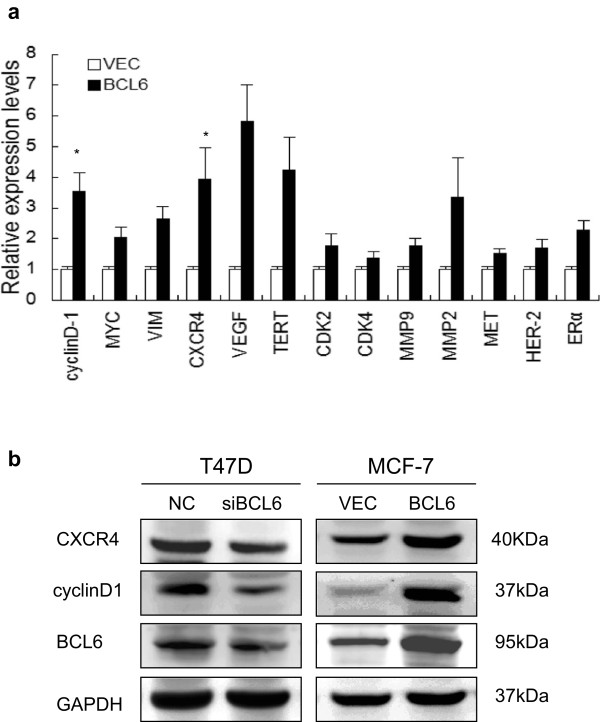
**Effects of BCL6 expression on CXCR4 and cyclinD1 expression. (a)** qRT-PCR. MCF-7 cells were transiently transfected with BCL6 cDNA or negative control vector and grown for 2 days. **(b)** Western blot. MCF-7 and T74D cells were transiently transfected with BCL6 cDNA, BCL6 siRNA, or negative control vector and grown for 2 days and subjected to Western blot. *, *P* < 0.05.

Thus far, we have demonstrated the effects of BCL6 expression in breast cancer. We next determined the possible underlying mechanism. In our study, we analyzed the expression levels of key genes involved in cell proliferation, survival and metastasis [20-23] after transfection with BCL6 cDNA or control by qRT-PCR and observed that expression of BCL6 increased cyclinD1 and CXCR4 mRNA expression in MCF-7 cells (Figure 5a). Using western blot, we confirmed that expression of cyclinD1 and CXCR4 protein was increased by transfection of BCL6 cDNA in MCF-7 cells, while depletion of BCL6 expression decreased their expression in T47D cells (Figure 5b), suggesting that BCL6 might regulate expression of these oncogenes.

### BCL6 is a direct target of miR-339-5p

**Figure 6:**
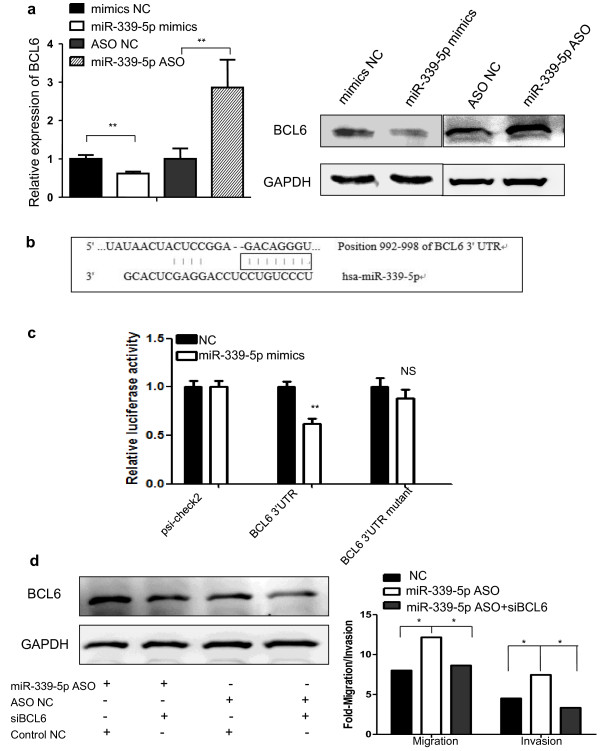
**BCL6 as the direct target gene of miR**-**339**-**5p in breast cancer cells. (a)** qRT-PCR and Western blot. miR-339-5p mimics or miR-339-5p ASO was transiently transfected into T47D cells and subjected to analysis of BCL6 expression. **(b)** The binding site of BCL6 3′-UTR and miR-339-5p. **(c)** Luciferase reporter assay. T47D cells were transfected with psiCHECK-2-BCL6 3′-UTR or psiCHECK-2-BCL6 mutated 3′-UTR plus either miR-339-5p mimics or negative control and subjected to luciferase reporter assay. **(d)** Tumor cell migration and invasion assay and Western blot. MCF-7 cells were grown and transiently transfected with miR-339-5p ASO, miR-339-5p ASO plus BCL6 siRNA or scrambled sequence oligonucleotides as negative control for 2 days and subjected to migration, invasion and western blot assays. *P < 0.05; **P < 0.01.

Our previous study revealed that expression of BCL6 was down-regulated by miR-339-5p [18]. To verify BCL6 as the bona fide target of miR-339-5p, qRT-PCR and western blot analyses were performed to detect the expression levels of BCL6 in breast caner cells transfected with either miR-339-5p mimics or miR-339-5p ASO. Expression of miR-339-5p resulted in substantial reduced levels of BCL6 mRNA and protein in T47D cells, while depletion of miR-339-5p expression using miR-339-5p ASO significantly increase in levels of BCL6 mRNA and protein (Figure 6a). Bioinformatic analysis utilized the algorithm of Targetscan [24] showed that BCL6 mRNA contains a 3’-UTR element complementary to the miR-339-5p binding site. Forced expression of miR-339-5p reduced the activity of a luciferase reporter gene fused to the full length wild-type BCL6 3’UTR, indicating that miR-339-5p directly targets BCL6 (Figures 6b and c).

A previous study has reported that expression of miR-339-5p inhibited breast cancer cell migration and invasion [18]. We therefore proceeded to determine whether BCL6 was involved in miR-339-5p-mediated cell migration and invasion. We first depleted BCL6 expression by siRNA and co-transfected the cells with miR-339-5p ASO and observed that depletion of BCL6 expression significantly abrogated miR-339-5p ASO-induced tumor cell migration and invasion, indicating that BCL6 plays a critical role at the downstream of miR-339-5p (Figure 6d).

## Discussion

In the current study, we first detected BCL6 expression in breast cancer vs. breast benign disease tissue specimens and found that levels of BCL6 mRNA and protein were significant higher in breast cancer tissues than in breast benign disease tissues. Expression of BCL6 protein was associated with tumor size, lymph node metastasis, advanced clinical stages, higher tumor grade and also Ki67 labeling index in breast cancer. Moreover, Kaplan-Meier analyses showed the association of BCL6 protein expression with poor overall and relapse-free survivals of patients. After that, we assessed the effects of forced expression or depletion of BCL6 protein on breast cancer cell viability, apoptosis, migration, invasion and gene expression *in vitro* and in nude mice. We found that expression of BCL6 increased tumor cell viability, migration, invasion, and survival as well as expression of cyclinD1, and CXCR4 *in vitro*. BCL6 expression also induced formation and growth of xenografts in nude mice. The bioinformatic analysis and luciferase assay showed that BCL6 expression could be directly targeted by miR-339-5p. In conclusion, our current study demonstrated that the reduced miR-339-5p expression [our previous data (ref)] promoted BCL6 expression, which in turn induces cyclinD1 and CXCR4 expression for induction of breast cancer cell proliferation and invasion. Future studies will investigate whether target of BCL6 expression could be useful as a novel therapeutic strategy for breast cancer.

Clinically, patients with early stage breast cancer have relatively high survival rates, but most of breast cancer still progress unnoticeably and lead to 30% of patients relapse with a distant metastatic disease [25]. For past several decades, numbers of biomarkers, including human epidermal growth factor receptor 2 (HER-2/neu), estrogen receptor (ER) and progesterone receptor (PR), have been identified and routinely used in clinic to predict prognosis and therapeutic response of the patients [26,27]. In the current study, we showed BCL6 as a potential biomarker to predict overall and disease-free survival of breast cancer patients. Indeed, BCL6 protein has been comprehensively accepted to play a role in supporting hematologic cell neoplastic transformation. Overexpression of BCL6 has been observed in 40% of DLBCL [28], 14% of follicular lymphomas [29], and 48% of nodular lymphocyte-predominant Hodgkin lymphomas [30]. BCL6 expression has also been observed in skin squamous cell carcinoma [9], neuroblastoma [10], melanoma [31], and breast cancer [12-14]. In breast cancer, Logarajah et al. showed that BCL6 protein was expressed in most of histologically high-grade ductal breast carcinoma compared with low-grade tumors [12]. Bos et al. reported that expression of BCL6 protein was significantly higher in breast cancer than in normal breast tissues [13]. Another most recent study showed that high expression of BCL6 protein associated with unfavorable clinical outcome of ER-positive and premenopausal breast cancer patients [32]. Our current data are consistent with these published studies [12,13] by confirmed the oncogenic effect of BCL6 in breast cancer, similar to those in hematologic malignancies.

However, Pinto et al. showed that expression of BCL6 protein was significantly down-regulated in metastatic lymph nodes than in the corresponding primary breast cancer [14]. The reason for this discrepancy is unknown, but led us to studying the effects of BCL6 on breast cancer cell lines. Using gain and loss-of-function approaches, our in vitro data demonstrated that expression of BCL6 did enhance breast cancer cell viability, anchorage-independent growth, migration, and invasion capacity. Concordantly, expression of BCL6 promoted low-invasive MCF-7 cell growth and invasiveness in vivo. These data demonstrated that BCL6 is an oncogene or has oncogenic effects on breast cancer cells. These data were consistent with a previous study showing that BCL6 expression prevented a mammary epithelial cell line to form the ducts and apoptosis [12]. However, it remains unknown how BCL6 contribute to breast cancer development or progression.

Thus, we then explored the underlying molecular events. CXCR4 has been identified as an important chemokine receptor in regulating cell migration, invasion and metastasis in breast cancer [23]. CyclinD1 is an essential cell cycle regulator for progression through G1 phase and is a candidate proto-oncogene [33]. We analyzed expression of CXCR4 and cyclinD1 in BCL6 expressed or knocked down breast cancer cell lines and found that expression of these genes was all up-regulated by BCL6 expression. Moreover, our previous study revealed that expression of BCL6 was regulated by miR-339-5p [18], but it was unclear whether BCL6 is a direct target gene of miR-339-5p. Our luciferase assay indeed confirmed that BCL6 is the target gene of miR-339-5p. miRNAs are a class of non-protein-coding RNAs and possess an important function in regulation of gene expression [34]. By regulating their target mRNAs for direct degradation or inhibition of translation, miRNAs play important roles in many biological processes and altered miRNA expression contributes to cancer development and progression [34]. miR-339-5p has been implicated in regulation of cell proliferation, migration and invasion in different cancers [18,35]. Although miR-339-5p may target different genes, BCL6 is definitely one of them. Our current data on manipulating miR-339-5p expression did alter effects of BCL6 on breast cancer cells.

## Conclusions

Our current study demonstrated that high expression of BCL6 associated with tumor size, lymph node metastasis, advanced clinical stages, higher tumor grade, and Ki67 labeling index as well as poor prognosis of breast cancer. Manipulation of BCL6 expression using the gain and loss of function approaches affected breast cancer cell viability, apoptosis, migration, invasion and gene expression *in vitro* and in nude mice. At the gene level, BCL6 induced CXCR4 and cyclinD1 expression and BCL6 is the target gene of miRNA, miR-339-5p. Future studies will further investigate the underlying molecular mechanisms by which BCL6 is overexpressed in breast cancer and BCL6 expression contributes to breast cancer progression. In addition, we will also determine whether BCL6 is a potential biomarker for prediction of breast cancer prognosis or as a therapeutic target for breast cancer patients.

## Abbreviations

ASO: Antisense oligonucleotide; CDK2: Cyclin-dependent kinase 2; CDK4: Cyclin-dependent kinase 4; ERα: Estrogen receptor alpha; GAPDH: Glyceraldehyde-3-phosphate dehydrogenase; HER2: Human epidermal growth factor receptor; ISH: *in situ* hybridization; IHC: Immunohistochemistry; MMP2: Matrix metallopeptidase 2; MMP9: Matrix metallopeptidase 9; OS: Overall survival; PBS: Phosphate-buffered saline; qRT-PCR: Quantitative reverse transcription polymerase chain reaction; RFS: Relapse free survival; siRNA: Small interfering RNA; TERT: Telomerase reverse transcriptase; VIM: Vimentin; VEGF: Vascular endothelial growth factor.

## Competing interests

The author’s declare that they have no competing interests.

## Authors’ contributions

QW, XL, HY, YHH, SY, XWC, GLZ and XJK performed experiments and summarized the data; WYW, XNW, ZT, PEL, and ZSW designed experiments; XCX and ZSW drafted the manuscript and critically discussed the data and manuscript; all authors have read and approved the final manuscript.

## Pre-publication history

The pre-publication history for this paper can be accessed here:

http://www.biomedcentral.com/1471-2407/14/418/prepub

## Supplementary Material

Additional file 1: TableS1 Sequences of miRNA and siRNA oligonucleotides. **Table S2.** The sequence of the oligonucleotide primers used for real-time PCR are as follows. Click here for file

Additional file 2: Figure S1 Modulation of the expression of BCL6 in brest cancer cell lines *in vitro*. **(a)** BLC6 siRNA was transiently transfected into T47D cells to decrease the expression of BLC6. **(b)** BLC6 cDNA was transiently transfected into MCF-7 cells to increase the expression of BCL6.**, P< 0.01. Click here for file
