# Supplementary material for: B-cell lymphoma 6 protein stimulates oncogenicity of human breast cancer cells
Source: BMC Cancer. 2014 Jun 10;14:418. doi: 10.1186/1471-2407-14-418 (PMC4065600; doi:10.1186/1471-2407-14-418)
Supplement: Additional file 1: TableS1 — Sequences of miRNA and siRNA oligonucleotides. Table S2. The sequence of the oligonucleotide primers used for real-time PCR are as follows. [file 1471-2407-14-418-S1.doc]

**Supplemental** **Table1 Sequences of miRNA and siRNA oligonucleotides**

| miRNA and siRNA Duplexes | Sense Strand (5'-3') | Antisense Strand (5'-3') |
| --- | --- | --- |
| hsa-miR-339-5p mimic | 5’-UCCCUGUCCUCCAGGAGCUCAGGUGAGCUCCUGGAGGACAGGGAUU-3’ |  |
| mimic Negtive control | 5’-CAGUACUUUUGUGUAGUACAA-3’ |  |
| hsa-miR-339-5p ASO | 5’-CGUGAGCUCCUGGAGG ACAGGGA-3’ |  |
| ASO NC | 5’-UUCUCCGAACGUGUCACGUTT-3’ | 5’-ACGUGACACGUUCGUAGAATT-3’ |
| BCL6 siRNA | 5’-CGGCUCAAUAACAUCGUUATT-3’ | 5’-UAACGAUGUUAUUGAGCCGTT-3’ |
| Negative control | 5’-UUCUCCGAACGUGUCACGUTT-3’ | 5’-ACGUGACACGUUCGGAGAATT-3’ |

**Supplemental Table 2** **The sequence of the oligonucleotide primers used for real-time PCR are as follows:**

| Gene | Forward primer | Reversed primer |
| --- | --- | --- |
| BCL6 | CCAGCCACAAGACCGTCCAT | CTCCGCAGGTTTCGCATTT |
| CDK2 | GCTAGCAGACTTTGGACTAGCCAG | AGCTCGGTACCACAGGGTCA |
| CDK4 | CTGGTGTTTGAGCATGTAGACC | AAACTGGCGCATCAGATCCTT |
| CXCR4 | GGGCCTGAGTGCTCCAGTAG | GGGTAGAAGCGGTCACAGAT |
| CyclinD1 | GGTGGCAAGAGTGTGGAG | CCTGGAAGTCAACGGTAGC |
| ERα | ACCTTCTAGAATGTGCCTGGCTAG | CTCATGCCAGGCACATTCTAGAAG |
| GAPDH | TGCACCACCAACTGCTTAGC | GGCATGGACTGTGGTCATGAG |
| HER-2 | ACCGGCACAGACATGAAGCT | AGGAAGGACAGGCTGGCATT |
| MiR-339-5P | GGGTCCCTGTCCTCCA | TGCGTGTCGTGGAGTC |
| MET | TGGTGCAGAGGAGCAATGG | CATTCTGGATGGGTGTTTCCG |
| MMP-2 | CAAAAACAAGAAGACATACATCTT | GCTTCCAAACTTCACGCTC |
| MMP-9 | TGGGGGGCAACTCGGC | GGAATGATCTAAGCCCAG |
| TERT | GGAGCAAGTTGCAAAGCATTG | TCCCACGACGTAGTCCATGTT |
| U6 | GCTTCGGCAGCACATATACTAAAAT | CGCTTCACGAATTTGCGTGTCAT |
| Vimentin | CCTTGAACGCAAAGTGGAATC | GACATGCTGTTCCTGAATCTGAG |
| VEGF | CGAAGTGGTGAAGTTCATGGATG | TTCT-GTATCAGTCTTTCCTGGTGAG |
