# Supplementary material for: B-cell lymphoma 6 protein stimulates oncogenicity of human breast cancer cells
Source: BMC Cancer. 2014 Jun 10;14:418. doi: 10.1186/1471-2407-14-418 (PMC4065600; doi:10.1186/1471-2407-14-418)
Supplement: Additional file 2: Figure S1 — Modulation of the expression of BCL6 in brest cancer cell lines in vitro. (a) BLC6 siRNA was transiently transfected into T47D cells to decrease the expression of BLC6. (b) BLC6 cDNA was transiently transfected into MCF-7 cells to increase the expression of BCL6.**, P< 0.01. [file 1471-2407-14-418-S2.ppt]

## Slide 1
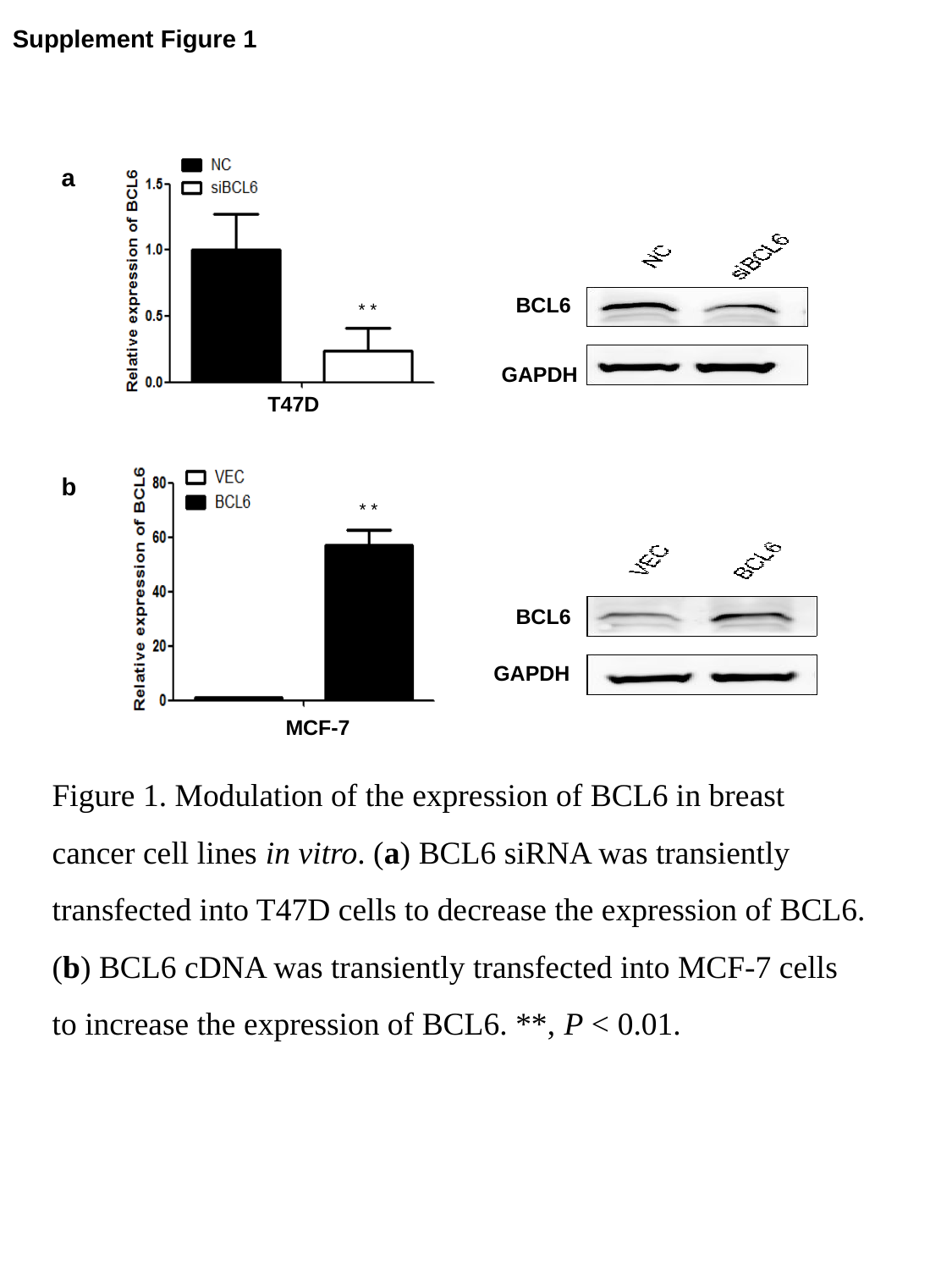

Supplement Figure 1
a
BCL6
GAPDH
* *
T47D
b
* *
BCL6
GAPDH
MCF-7
Figure 1. Modulation of the expression of BCL6 in breast cancer cell lines in vitro. (a) BCL6 siRNA was transiently transfected into T47D cells to decrease the expression of BCL6. (b) BCL6 cDNA was transiently transfected into MCF-7 cells to increase the expression of BCL6. **, P < 0.01.
